# Hemophagocytic Lymphohistiocytosis: Management and Special Consideration in Human Immunodeficiency Virus Positive Patients with Immune Reconstitution Syndrome

**DOI:** 10.7759/cureus.5402

**Published:** 2019-08-16

**Authors:** Bikramjit S Bindra, Katherine Garcia de de Jesus, Oscar Cisneros, Vinicius M Jorge, Harpreet Kaur

**Affiliations:** 1 Internal Medicine, Government Medical College and Hospital, Chandigarh, IND; 2 Internal Medicine, St. Barnabas Hospital Health System / Albert Einstein College of Medicine, Bronx, USA; 3 Hematology and Medical Oncology, Albert Einstein Medical Center, Philadelphia, USA; 4 Internal Medicine, Albert Einstein Medical Center, Philadelphia, USA

**Keywords:** hlh, iris, hemophagocytic lymphohistiocytosis

## Abstract

The human body is capable of reacting to multiple aggressors by developing an inflammatory response with the secretion of inflammatory cytokines. The worrisome clinical manifestations occur when this inflammatory response is disproportionate. Hemophagocytic lymphohistiocytosis (HLH) is a rare and severe condition characterized by an overwhelming inflammatory response that may result in end-organ damage and might be fatal. Correspondingly, immune reconstitution inflammatory syndrome (IRIS) is another well-known disorder, seen commonly in human immunodeficiency virus (HIV)-infected patients after the commencement of highly active antiretroviral therapy (HAART). Both entities share a similar clinical presentation and a dismal prognosis. Due to widespread clinical manifestations and laboratory abnormalities, diagnosis is often missed at the time of presentation. There is little consensus on the treatment of secondary HLH, which is usually handled on a case-by-case basis. Rapid curbing of the widespread inflammatory response is the main goal of treatment. To the best of our knowledge, there is scarce literature available on the coexistence of HLH and IRIS; therefore, medical management in the co-occurrence of these two conditions needs to be further investigated.

## Introduction and background

Hemophagocytic lymphohistiocytosis (HLH) represents a life-threatening condition that results from a schematized process ensuing in hyperproduction of cytokines, that leads to fatal end-organ damage. HLH has been linked with multiple conditions, such as infectious diseases, malignancies, and autoimmune diseases. The clinical picture is vague, including fever, hepatomegaly, splenomegaly, and pancytopenia. The lack of specific manifestations poses a challenge in establishing a definitive diagnosis. However, encompassing a mortality of about 95%, launching a timely diagnosis is a critical challenge for physicians [1,2]. Interestingly, the above-mentioned definition of HLH also fits the description of another well-known entity, immune reconstitution inflammatory syndrome (IRIS), in which immune system rebuilding after initiation of highly active antiretroviral therapy (HAART) results in an exaggerated inflammatory response [3,4]. This review is intended to describe the challenges faced during the diagnosis and treatment of HLH in the setting of IRIS in human immunodeficiency virus (HIV) positive patients. 

Pathophysiology

HLH is usually a disproportionate response to an inflammatory signal that results in hypercytokinemia. This excessive response is thought to be due to some form of impairment in the function of cytotoxic T-lymphocytes (CTLs) and natural killer (NK)/T-cell function. Histopathologically, HLH is characterized by a prominent and diffuse accumulation of lymphocytes and mature macrophages, which occasionally exhibit hemophagocytosis (phagocytosis by macrophages of erythrocytes, leukocytes, platelets, and their precursors). Two distinct forms of HLH have described in the existing literature. The primary or genetic HLH is a fatal disorder found in infants and young children, predominantly due to mutations in genes responsible for the cytotoxic function of NK cells and T-lymphocytes [1,2]. The secondary/acquired form of HLH is found mostly in adults and is often associated with infections, malignancies, autoimmune diseases and acquired immunodeficiency states like acquired immunodeficiency syndrome (AIDS), transplantation, chemotherapy or other forms of immunosuppressive treatment as shown in Table 1 [5,6].

**Table 1:** Etiologies of acquired HLH Abbreviations: Epstein-Barr virus - EBV, Cytomegalovirus - CMV, Human Herpesvirus-8 - HHV-8, Herpes simplex viruses - HSV, Hepatitis A Virus - HAV, Hepatitis B virus - HBV, Hepatitis C Virus - HCV, Species - spp., Mycobacterium avium complex - MAC, Natural killer cell  - NK- cell, Anaplastic Large Cell Lymphoma - ALCL, Acute lymphoblastic leukemia - ALL, Systemic onset Juvenile Idiopathic Arthritis - SoJIA, Systemic lupus erythematosus - SLE, Acquired immunodeficiency syndrome - AIDS

1. Infections:
-Viral: Herpesviruses (EBV, CMV, HHV-8, HSV), HIV, HTLV, Adenovirus, HAV, HBV, HCV, Measles, Mumps, Rubella, Dengue, Hantavirus, Parvovirus B19, Enterovirus, Influenza virus
-Bacterial: Staphylococcus aureus, Campylobacter spp., Fusobacterium spp., Mycoplasma spp., Chlamydia spp., Legionella spp., Streptococcus pneumoniae, Salmonella typhi, Rickettsia spp, Brucella spp, Ehrlichia spp, Borrelia burgdorferi
-Mycobacterial: Mycobacterium tuberculosis, MAC, Mycobacterium kansasii
-Parasitic: Plasmodium falciparum, Plasmodium vivax, Toxoplasma spp., Babesia spp., Strongyloides spp., Leishmania spp.
-Fungal: Candida spp., Cryptococcus spp., Pneumocystis spp., Histoplasma spp., Aspergillus spp., Fusarium spp.
2. Hematological malignancies: T-cell/NK-cell lymphomas, ALCL, ALL, Hodgkin’s lymphoma, multiple myeloma, acute erythroid leukemia
3. Non-hematological malignancies: Prostate cancer, lung cancer, hepatocellular carcinoma
4. Autoimmune diseases: SoJIA, adult-onset Still's disease, SLE, Kawasaki disease, seronegative spondyloarthropathies
5. Acquired immunodeficiency states: AIDS, transplantation, chemotherapy, other immunosuppressive treatments

After the initiation of HAART, HIV infected patients start a process of replenishment of the immune cells. This process, especially in very immunodeficient patients, may result in a disproportionate and exaggerated host inflammatory response, similar to the one observed in HLH [7]. Risk factors associated with the development of IRIS include severe immunodeficiency, patient’s comorbidities, antigen burden, and rapid immune response to HAART [8]. Although immunopathogenesis of IRIS is still uncertain, factors like quantitative and qualitative improvements in CD4 cells and shift of balance between Th-1 and Th-2 responses leading to an increase in IL-2, IFN-γ, IL-6, and soluble IL-6 receptors, are thought to play a significant role [9]. Additionally, the high level of macrophage colony-stimulating factor produced by T-cells is capable of stimulating monocytes/macrophages leading to downhill production of TNF-α and IL-6 [10].

Diagnosis

Diagnostic guidelines for HLH were revised by the HLH Study Group of the Histiocyte Society, suggesting that the diagnosis of HLH can be made on two bases-either a molecular diagnosis consistent with HLH or if the patient meets five of the following eight criteria: fever, splenomegaly, at least two lineages of cytopenia, hypertriglyceridemia (≥265 mg/dL) or hypofibrinogenemia (≤1.5 g/L), hemophagocytosis on histology, low/absent NK-cell activity, hyperferritinemia (≥500 μg/L) and increased levels of soluble interleukin-2 receptor (sCD25), a marker of macrophage activity) [11]. Diagnostic guidelines have been summarized in Table 2. These criteria are non-specific and sometimes manifest late in the illness course.

**Table 2:** Diagnostic criteria for HLH

A. Initial diagnostic criteria (to be evaluated in all patients with suspected HLH)
-Fever (>38.5 C)
-Splenomegaly
-Cytopenias (at least two lines of peripheral blood involved): Anemia Hb <9d/dL, thrombocytopenia <1000/μL, neutropenia <1000/ μL
-Hypertriglyceridemia and/or hypofibrinogenemia: Fasting triglycerides ≥3.0 mmol/L (i.e., ≥265 mg/dL) and/or Fibrinogen ≤1.5 g/L
-Hemophagocytosis in bone marrow, spleen, lymph nodes, no evidence of malignancy.
B. New diagnostic criteria:
-Decreased or absent natural killer-cell activity.
-Ferritin ≥500 μg/L
-sCD25 (soluble interleukin - 2 receptor) ≥2400 U/mL or ≥4800 pg/mL)
Comments: If hemophagocytic activity is not proven at the time of presentation, further search for hemophagocytic activity is encouraged. If the bone marrow specimen is not conclusive, material may be obtained from other organs. Serial marrow aspirates over time may also be helpful. The following findings may provide strong supportive evidence for the diagnosis:
a. Spinal fluid pleocytosis (mononuclear cells) and/or elevated spinal fluid protein,
b. Histological picture in the liver, resembling chronic persistent hepatitis (on biopsy)

## Review

HLH occurs more frequently in the HIV/AIDS population and has a septic-shock like presentation [12]. It has been shown that HLH in advanced AIDS is frequently driven by an underlying process (secondary HLH). Fardet et al. described a large case series (58 patients) of HLH in the setting of HIV which revealed that hematological malignancy accounted for 55% of cases while underlying infections accounted for 41% of cases. Among the most commonly encountered etiologies were Hodgkin’s lymphoma, tuberculosis, other B-cell lymphomas, and multicentric Castleman’s syndrome (MCD) [13]. On perusal of another large retrospective case series of 162 HIV and non-HIV-infected patients with HLH, we found that underlying hematological malignancy (56.8% of cases) was more common than infection (40% of cases). Again, Hodgkin’s lymphoma, B-cell lymphoma, and MCD were the most common non-infectious etiologies. The infectious etiologies included bacteria, mycobacteria, viruses such as Epstein-Barr virus (EBV) and Cytomegalovirus (CMV), and parasitic causes like toxoplasmosis, leishmaniasis, Plasmodium falciparum, Pneumocystis jirovecii and histoplasmosis [14]. If we only consider the viral etiologies, EBV emerges as a much more common trigger of HLH syndrome as compared to Human Herpesvirus-8 (HHV-8) [15,16].

To the best of our knowledge, there are only a handful of cases of HHV-8-related HLH. They included both HIV-positive and HIV-negative patients as well as post-renal transplant patients. In some of the instances, HHV-8 related HLH occurred in the setting of underlying MCD, Kaposi sarcoma (KS) or primary effusion lymphoma (PEL), while in others, HHV-8 infection resulted in HLH in the absence of any underlying malignancy [17-20]. It is also evident that HHV-8 viral load correlates with disease activity [17]. Over half of these patients died despite treatment due to delays in diagnosis.

The treatment strategy of HLH involves treating the underlying cause to halt the overactivation of the immune system. Infectious causes and underlying malignancies must be investigated to direct disease-specific treatment. Rituximab can be initiated if the EBV infection is suspected. Intravenous immunoglobulin (IVIG) is considered an appropriate addition therapy for most viral infections [20]. If the patient is stable, treatment of the underlying cause along with steroids (to curb the widespread inflammation) and close follow up might be appropriate. However, most patients are critically ill, and aggressive treatment should be provided as soon as possible without waiting for the results. Etoposide became the standard of care after being tested in large studies and shown to improve clinical outcomes [21]. Etoposide acts via a cytolytic effect on dividing T-cells, leading to ineffectively responding T-cells; thus, diminishing the cytokines in circulation [22]. In 1994, a large prospective study in the pediatric population (HLH-94) was performed. Treatment regimen included 8-week induction with dexamethasone and etoposide; intrathecal methotrexate was given if neurologic symptoms were present. Early relapses were observed, and it was followed by another, relatively newer study (HLH-2004), that included the addition of cyclosporine during the induction phase [21].

According to the HLH-2004 protocol, an 8-week course consisting of three drugs-etoposide, dexamethasone, and cyclosporine-is recommended as a part of the “initial therapy.” The supportive therapy includes prophylactic cotrimoxazole, consideration of antiviral therapy in patients with ongoing viral infections, and IVIG. Allogenic bone marrow transplantation is recommended for patients with a familial disease/molecular diagnosis and those refractory to first-line therapy [11]. However, it is not recommended to perform a bone marrow transplantation in the setting of an active disease due to a high risk for graft versus host disease. Fischer et al. were the first to describe a cure for pediatric patients with primary HLH, which became the mainstay of treatment for both children and adults [23]. There is no consensus regarding the treatment for secondary HLH. Cytotoxic or immunosuppressive treatment is not required in all cases. Chemotherapy-related toxicity can add more burden to the critically ill patient, and a trial of dexamethasone might be appropriate [24]. Dexamethasone and etoposide dose frequency needs to be increased if disease reactivation is suspected. A steady increase in the levels of serum ferritin, sCD25, sCD163, and worsening liver function and blood counts reflect a relapse of the disease activity. If no partial response is seen within two to three weeks of induction therapy, salvage treatment should be considered. The data available on salvage therapy is scarce; however, alemtuzumab has shown significant activity against refractory HLH [25]. The limited existing literature recommends continuing HAART in cases of IRIS associated with HLH [26]. However, HLH carries a worse prognosis when compared to IRIS, and without treatment, HLH is invariably fatal.

## Conclusions

The acquired or secondary form of HLH has multiple underlying etiologies like infections, malignancies, autoimmune diseases, and acquired immunodeficiency states like AIDS. The clinical picture is vague, including fever, hepatomegaly, splenomegaly, and pancytopenia. Lack of specific manifestations poses a challenge in establishing a definitive diagnosis. Existing literature is deficient when it comes to unique scenarios like the co-occurrence of HLH and IRIS. Since HLH carries a worse prognosis than IRIS, it might be beneficial to discontinue HAART temporarily in such cases.
